# The Perio-Resto Interface: In Vitro Comparison of Two Deep Margin Elevation Techniques on Surface Roughness, Marginal Adaptation and Material Integrity

**DOI:** 10.3390/dj14030161

**Published:** 2026-03-11

**Authors:** Pablo Cores Ziskoven, Dorothea Vogel, Sven Schumann, David Kiramira, Thanya Nguyen, Andreas M. Geyer, Jens Weusmann, James Deschner

**Affiliations:** 1 Department of Periodontology and Operative Dentistry, University Medical Center of the Johannes Gutenberg University Mainz, 55131 Mainz, Germanydavid.kiramira@unimedizin-mainz.de (D.K.); andreas.geyer@unimedizin-mainz.de (A.M.G.); jens.weusmann@unimedizin-mainz.de (J.W.); james.deschner@uni-mainz.de (J.D.); 2 Institute of Anatomy, University Medical Center of the Johannes Gutenberg University Mainz, 55131 Mainz, Germany; sven.schumann@uni-mainz.de

**Keywords:** deep margin elevation, proximal box elevation, restorative dentistry, supracrestal connective tissue attachment, dentogingival complex, periodontology

## Abstract

**Background/Objectives:** Deep subgingival proximal carious lesions present significant restorative and periodontal challenges, especially when approaching the supracrestal attachment (SA). This study compared two established deep margin elevation (DME) tecniques—the modified matrix technique (MMT) and the matrix-free “R2 technique” (R2T)—with respect to surface roughness, marginal adaptation, surface integrity, voids and excess adhesive material. **Methods:** Forty extracted human mandibular molars were prepared with standardized proximal cavities 2–3 mm below the cementoenamel junction (CEJ) and randomly assigned to two groups (*n* = 20 each). Group 1 received DME with the modified matrix technique; Group 2 was treated with the R2T. In both groups, a flowable bulk-fill composite was applied. Surface characteristics and marginal adaptation were evaluated using scanning electron microscopy (SEM) and laser profilometry. Qualitative scoring and quantitative measurements were performed. Statistical analysis was conducted using GraphPad Prism (version 10.02.0). **Results:** The modified matrix technique resulted in significantly smoother composite surfaces (*p* < 0.001), whereas the R2T showed significantly fewer voids, better marginal adaptation, and less excess bonding material (*p* < 0.05). No statistically significant difference was observed in surface integrity between the groups. **Conclusions:** While the MMT produced smoother surfaces, the R2T resulted in superior marginal quality with fewer voids and less excess adhesive material. The findings suggest technique-specific advantages rather than overall superiority, indicating that both approaches appear feasible. Clinical decision-making should be guided by anatomical and operator-related factors.

## 1. Introduction

Although the overall prevalence of caries is decreasing in industrialized countries such as Germany, deeply subgingivally destroyed teeth remain a significant problem both internationally and nationally [1]. Demographic changes have shifted the prevalence from children to adults and seniors [2,3]. Increased life expectancy and reduced tooth loss have led to a rise in root caries or deep proximal defects [1,4,5,6,7]. Representative bitewing radiographs from over 1000 patients revealed that a large proportion of lower and upper molars exhibited carious defects 5–7 mm in depth [8]. This trend is partly attributable to an increased risk of exposed root surfaces, i.e., recessions. These are associated with aging, combined with decreasing sensorimotor abilities that are essential for maintaining adequate oral hygiene [9]. Previous studies have demonstrated that caries progression in dentin occurs at nearly twice the speed of lesions confined to enamel [10,11]. Tooth loss invariably leads to functional limitations and diminished quality of life [12,13]. Given the advanced age of many patients, comorbidities are common, rendering oral surgical procedures, such as extractions, subject to considerable pre-, peri-, and postoperative challenges [14]. Preserving such teeth often helps to avoid riskier surgical interventions while conservatively maintaining function, phonetics, and aesthetics. Moreover, tooth preservation is generally more cost-effective for both patients and society compared to prosthetic replacements [15,16].

Reconstructing deeply subgingival defects presents multiple clinical challenges. Key prerequisites for the longevity of restorations—whether direct composite or indirect laboratory-fabricated—include contamination-free, controlled impression-taking, accurate fitting, and precise placement of the restorative materials. Contamination by saliva or blood may cause inaccuracies during analogue or digital impressions, potentially accumulating errors over the course of treatment. Furthermore, contamination can compromise dentin adhesive bonding [17,18,19]. Deep subgingival defects often hinder visual control during caries excavation and can irritate the proximal gingiva, which is frequently inflamed and prone to bleeding due to its proximity to the lesion. Such contamination of the visual and working field complicates the procedure considerably. Electric gingivectomy and hemostatic agents, such as ferric sulfate, can re-establish access, ensure a clean field, and facilitate impression-taking or digital scanning for indirect restorations. Laboratory fabrication requires several days, so restoration delivery follows a provisional phase of variable duration. During fitting and placement, clinicians encounter similar challenges: the deep subgingival margin complicates fit verification, while contamination-free placement and complete excess removal are nearly impossible. Residual cement and excess must, however, be meticulously removed to prevent tissue irritation [20,21,22].

Beyond technical and material limitations, clinicians must respect anatomical structures to avoid inducing persistent inflammation. The dento-gingival complex extending from coronal to apical comprises free gingiva, sulcular epithelium, junctional epithelium, and supracrestal connective tissue attachment [23,24]. Current evidence suggests that a smooth, polished restoration margin well adapted to the junctional epithelium does not necessarily cause irritation [25]. However, if the restoration margin extends into the 2–3 mm wide connective tissue attachment, it may cause periodontal inflammation, suppuration or recession, depending on the gingival phenotype [26,27]. Conventional treatments to restore physiological dentogingival dimensions include periodontal crown lengthening, orthodontic extrusion and intentional replantation, all of which are relatively invasive.

**Figure 1:**
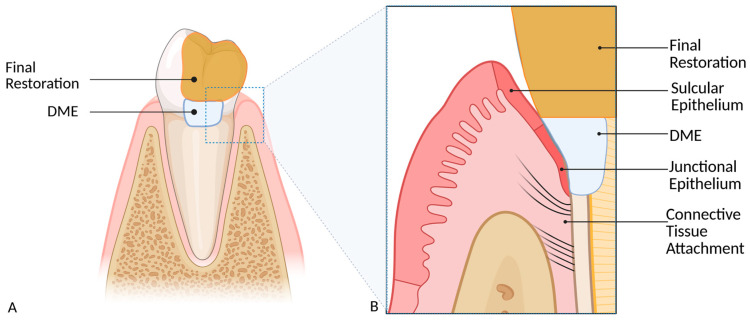
(**A**) Schematic representation of the location of the deep margin elevation (DME) as an intermediate layer between the final restoration and the cavity floor. (**B**) Close-up view of A showing the location of the DME in relation to the sulcus and junctional epithelium, as well as to the connective tissue attachment.

A less invasive alternative is deep margin elevation (DME) (Figure 1). In DME, the deep carious defect is carefully exposed and directly restored using acid-etching and composite. This approach elevates the cavity floor to a supragingival level, thus circumventing the aforementioned technical, material and biological challenges. The elevated margin facilitates rubber dam isolation, enabling contamination-free preparation, impression taking and, most importantly, restoration placement. Two DME techniques have been described in the literature: the “Modified Matrix Technique” (MMT) by Magne and Spreafico [28] and the matrix-free “R2 Technique” (R2T) by Frese et al. [29]. The primary difference lies in the use of a customized stainless steel matrix to shape the subgingival contour in the technique described by Magne and Spreafico, whereas the R2T is performed without a matrix. Since excess composite is unavoidable with R2T, unlike with MMT, the outer surface of the raised increment has to be meticulously contoured and polished freehand. The elevated proximal box can be further restored with either an adhesively bonded indirect all-ceramic restoration or a direct composite filling.

Both techniques are clinically established approaches for deep margin elevation. However, they are based on fundamentally different concepts of margin formation and contouring, which may have distinct implications for surface quality, marginal adaptation, and the presence of excess material in the subgingival connective tissue area. Since these parameters are critical for periodontal compatibility and long-term restorative success, a direct experimental comparison under standardized conditions is required to clarify potential technique-related differences. This study therefore aimed to analyze extracted teeth treated with either the MMT or the R2T with respect to surface integrity, roughness, void formation, and marginal seal in order to determine which of the techniques is more suitable in generating optimally smooth and adapted surfaces. The primary null hypothesis was therefore: DME technique does not influence surface roughness of the elevated composite increment. Secondary null hypotheses stated that there are no differences between the two techniques regarding marginal adaptation, surface integrity, and the occurrence of voids and excess bonding material.

## 2. Materials and Methods

**Figure 2:**
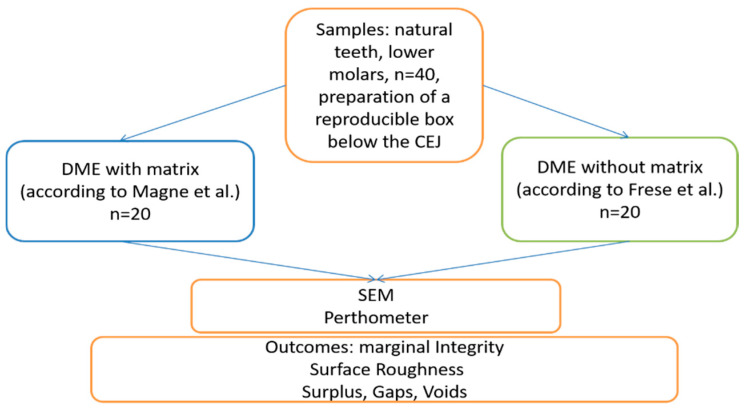
Experimental setup [29,30].

A schematic drawing of the experimental setup is shown in Figure 2. Extracted human mandibular molars were collected from patients undergoing routine dental treatment at the Department of Periodontology and Operative Dentistry of the University Medical Center Mainz. Teeth were extracted exclusively for therapeutic reasons, including advanced carious destruction, periodontal disease or lack of restorability. No teeth were extracted for the sole purpose of this study. All patients provided written informed consent allowing the use of biological surplus material for scientific purposes. No personal or clinical patient data were recorded or used for the study and the teeth were irreversibly anonymized prior to further processing. The study was conducted in accordance with the principles of the Declaration of Helsinki.

After extraction, teeth were visually inspected under magnification to exclude specimens with cracks, fractures, extensive restorations or structural defects. Only intact molars without visible damage to the crown or root structure were included. Extracted teeth were cleaned of residual soft tissue and stored in a 0.1% thymol solution at 4 °C and were utilized within 3 months of extraction. All restorative procedures were performed by a single operator specialized in restorative dentistry with more than 8 years of clinical experience. Based on an a priori sample size calculation for the primary endpoint surface roughness (Ra), a total of forty extracted human mandibular molars were prepared (Diamond Bur 842.314.014, Komet dental, Brasseler, Lemgo, Germany) with standardized mesial and distal cavities, positioned 2–3 mm apical to the cementoenamel junction (CEJ), measuring 5 mm in width and 2 mm in depth. All procedures were performed under 5-fold magnification (OPMI Pico, Zeiss, Jena, Germany) and copious cooling using water spray from the dental handpiece (T1 Line C200l, Dentsply Sirona, Bensheim, Germany). After cavity preparation, teeth were randomly assigned (1:1) to the MMT or R2T group using a computer-generated randomization sequence created with GraphPad Prism version 10.02.0 (GraphPad Software, San Diego, CA, USA). The allocation sequence was generated by a member of the research group who was not involved in the restorative procedures. Group assignment was concealed from the operator performing the restorations until the time of treatment. Each tooth constituted one experimental unit. To avoid pseudoreplication due to within-tooth correlation, all outcomes were analyzed at the tooth level (*n* = 20 teeth per group) by aggregating mesial and distal measurements per tooth. Etching was performed for 10 s with 35% phosphoric acid (DeTrey Conditioner 36, Dentsply Sirona, Bensheim, Germany), followed by a 15 s water rinse. A self-etching single-bottle adhesive (P&B Active, Dentsply Sirona, Bensheim, Germany) was gently applied with agitation for 10 s, then air-blown until no puddles or excess adhesive remained, ensuring an even and homogeneous coating. The defects were built up in a 3 mm increment using a flowable bulk-fill composite (SDR, Dentsply Sirona, Bensheim, Germany) to ensure adequate light transmission at greater depths. Light curing was performed using an LED curing device (Smart lite Pro, Dentsply Sirona, Germany) with a light intensity of 1250 mW/cm^2^. The light source was positioned coronally to the defect at a distance of approximately 6 mm perpendicular to the composite surface. Light curing was deliberately not performed directly on the composite surface or at a very close distance, as positioning the light source in direct contact with the composite surface seems unrealistic in the clinical setting of DME. The increment was polymerized for 40 s to compensate for the increased curing distance, as it is advised in a clinical setting [31,32]. To perform DME in the MMT group, a conventional circumferential stainless steel Tofflemire matrix band (Hawe Tofflemire Matrices, 0.03 mm; Kerr Corporation, Brea, CA, USA) was used. As part of this technique, the matrix band was trimmed as described in the original publication and adapted to the proximal box to achieve close marginal adaptation [28].

**Figure 3:**
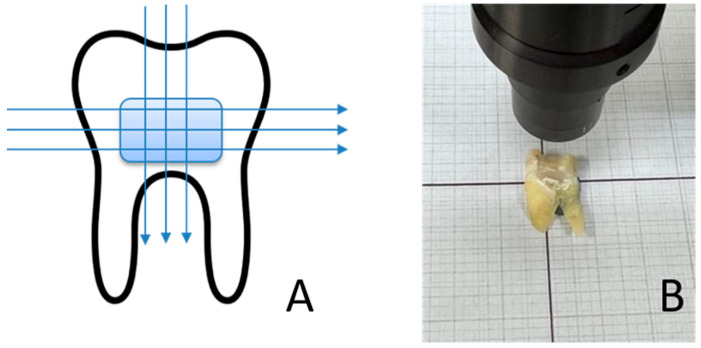
Surface scanner (**A**) Schematic representation of the measurement paths of the optical surface scanner. (**B**) Example of the experimental setup.

**Figure 4:**
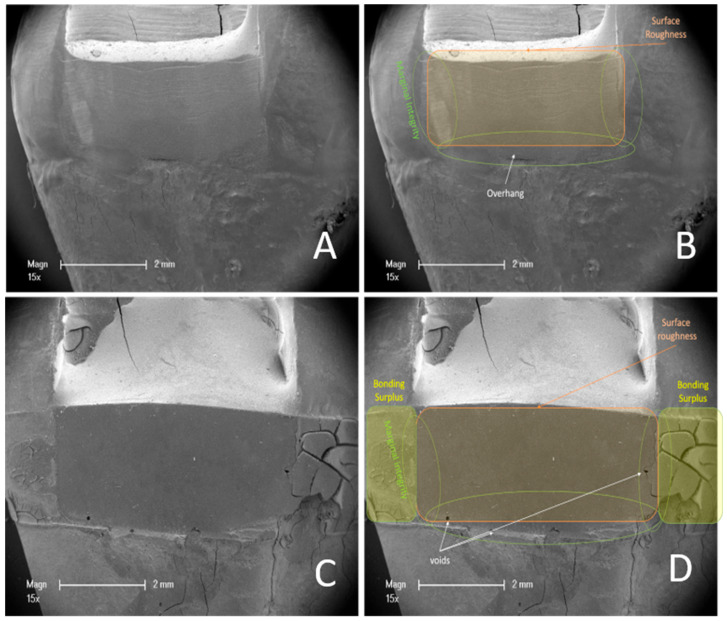
SEM images (**A**) Representative SEM image of a tooth on which the DME was performed using the R2T. (**B**) Same tooth as in A with the areas to be analyzed marked: orange: surface roughness & integrity, green: marginal seal, white: overhang. (**C**) Representative SEM image of a tooth on which DME was performed using a modified matrix technique. (**D**) Same tooth as in (**C**) with the areas to be analyzed marked: orange: surface roughness & integrity, green: marginal seal, white: bubbles & air pockets, yellow: excess bonding material.

In the R2T group, restorations were built up freehand and contoured for 10 s with a conical Arkansas stone (Komet dental, Brasseler, Lemgo, Germany), then polished for 10 s each with a rubber polisher (Enhancer, Dentsply Sirona, Bensheim, Germany) and a silicon carbide-coated brush (Komet dental, Brasseler, Lemgo, Germany), as described in the original Paper [29]. The polishing time of 10 s per instrument was selected to standardize the finishing procedure across all specimens and to reflect a clinically realistic polishing duration in deep proximal areas. After 48 h of water storage, the teeth were separated in the sagittal plane using a diamond cutting disc (Komet dental) to create a flat contact surface (Figure 3B). The restored mesial and distal DME surfaces of each tooth were scanned using an optical surface scanner (Mahr Perthometer, Optical Scanner LS 10, Göttingen, Germany) along three horizontal and three vertical scan lines (Figure 3A). For each surface, six measurements (three horizontal and three vertical scan lines) were obtained and averaged to generate one Ra value per surface. The mesial and distal Ra values were subsequently averaged to obtain one Ra value per tooth for statistical analysis. The measurements were performed in triplicate. The measuring device was calibrated in advance using a calibration standard (Halle, Präzisions-Kalibriernormale, Edemissen, Germany). After determining the roughness, the teeth were analyzed using a scanning electron microscope (SEM) (ESEM XL 30, Philips, Eindhoven, The Netherlands) to assess various outcomes. The specimens were mounted on sample plates using a 25 mm guide tab. The samples were then sputtered with gold (Fine Vacuum Coater EM ACE 200, Leica, Wetzlar, Germany). The images were analyzed for visual roughness, surface integrity, overhangs and marginal fit. Scores were assigned for the electron microscope outcomes (Table 1). The areas covered by the scores are shown in representative images in Figure 4A–D. For statistical analysis, SEM scores obtained from mesial and distal surfaces were aggregated per tooth to generate one representative score per outcome and experimental unit. The tooth was defined as the experimental and statistical unit (*n* = 20 teeth per group) and measurements were aggregated at the tooth level prior to statistical testing. SEM images were evaluated independently by two blinded examiners. Prior to evaluation, both examiners were calibrated using representative reference images and predefined scoring criteria. Inter-rater reliability was calculated using Cohen’s kappa for binary outcomes and weighted Cohen’s kappa for the ordinal marginal seal score. In cases of disagreement, a consensus decision was reached through joint evaluation after the independent ratings had been completed. The measurements from the Perthometer and scores were evaluated using GraphPad Prism version 10.02.0 (GraphPad Software, San Diego, CA, USA). The data were tested for normality (Shapiro-Wilk test) and analyzed using an independent *t*-test (parametric) or Mann-Whitney U test (non-parametric), as appropriate. Chi-square tests were applied to compare categorical score data. Surface roughness was defined as the primary outcome. Secondary outcomes were adjusted for multiple testing using the Holm–Bonferroni correction. Effect sizes were calculated for all comparisons (Cliff’s δ for non-parametric comparisons, Cramér’s V for categorical data, and odds ratios (ORs) for contingency tables), and effect estimates are reported with corresponding 95% confidence intervals (95% CI). A significance level of *p* < 0.05 was considered statistically significant.

## 3. Results

**Figure 5:**
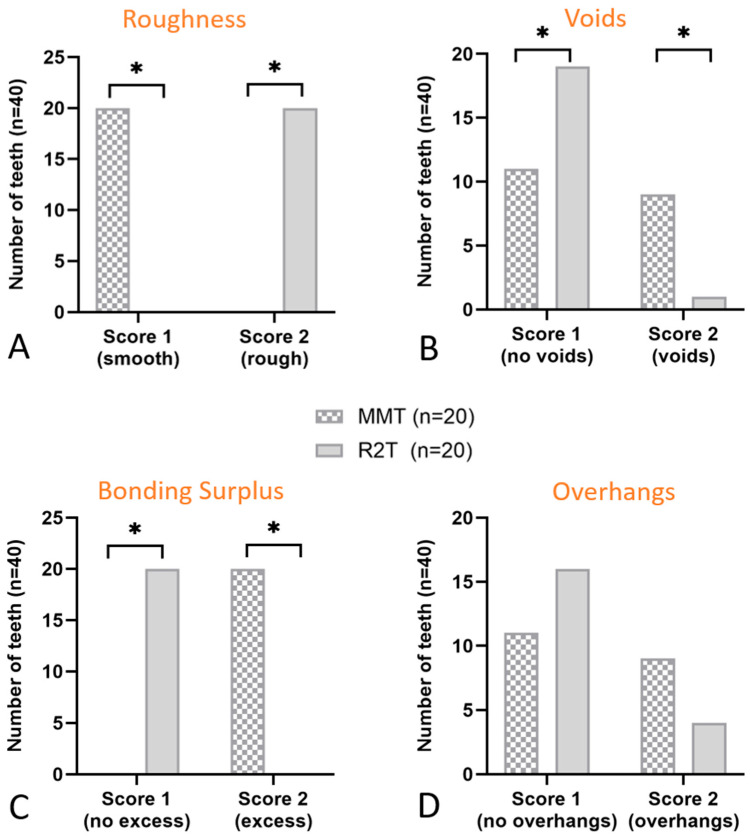
SEM evaluation. (**A**) Number of teeth that objectively had a visually smooth (score 1) or rough (score 2) surface. (**B**) Number of teeth that objectively had no bubbles or air pockets (score 1) or visible voids (score 2). (**C**) Number of teeth that objectively showed excess bonding (score 2) or none (score 1). (**D**) Number of teeth that objectively showed overhangs (score 2) or none (score 1). Analysis performed per tooth (*n* = 20 per group), after aggregation of mesial and distal surfaces. * significant (*p* < 0.05) difference between groups.

**Figure 6:**
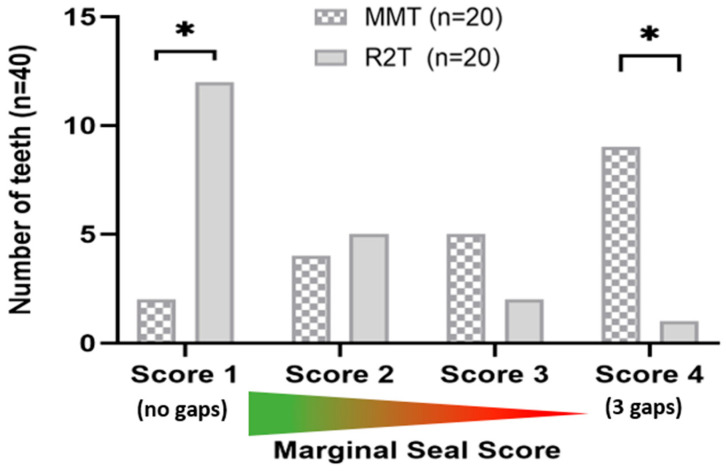
Distribution of SEM scores per tooth (*n* = 20 per group) that objectively showed 3 (score 1), 2 (score 2), 1 (score 3), or 0 (score 4) transition areas without edge gaps. Analysis performed per tooth (*n* = 20 per group), after aggregation of mesial and distal surfaces. * significant (*p* < 0.05) difference between groups.

**Figure 7:**
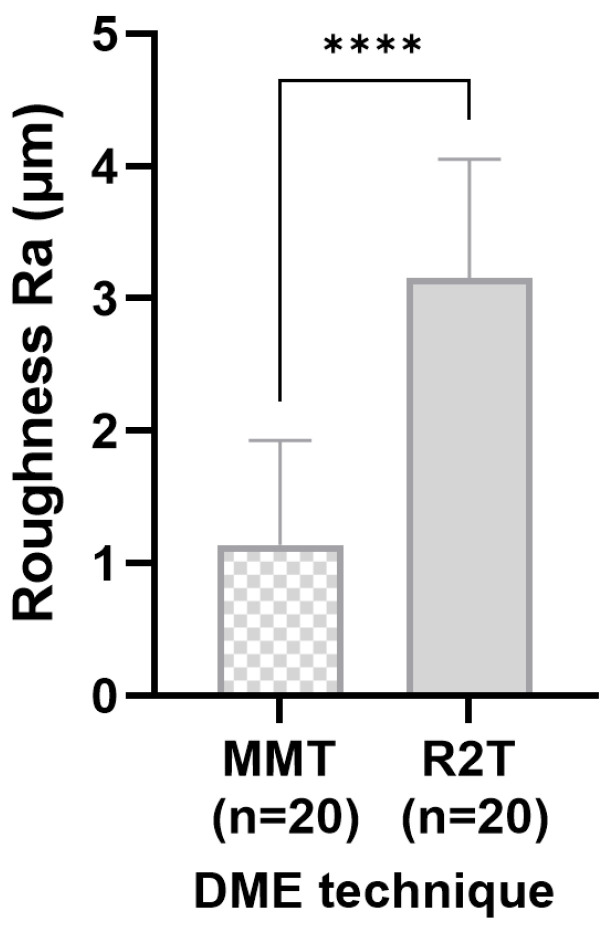
Surface roughness (Ra) measured using laser profilometry (in µm) of the teeth examined with matrix (left) and without matrix (right). Analysis performed per tooth (*n* = 20 per group), after aggregation of mesial and distal surfaces. **** significant (*p* < 0.05) difference between groups.

Evaluation of the scoring system revealed that the R2T produced visually rougher surfaces in all specimens examined (corrected OR ≈ 0.001, 95% CI; *p* < 0.001) (Figure 5A). This finding was confirmed quantitatively using surface scanning. Significantly fewer air inclusions or bubbles were observed with the R2T (V = 0.40; 95% CI; *p* = 0.011) (Figure 5B). No excess bonding material was detected in the R2T group (corrected OR ≈ 0.001, 95% CI; *p* < 0.001) (Figure 5C), whereas excess material was present in all specimens restored using the MMT (Figure 5C). The techniques did not significantly affect the formation of overhangs (OR = 3.27, 95% CI; *p* = 0.177), although a slight trend favoring the R2T was observed (Figure 5D). Marginal gaps occurred significantly more often in teeth restored with a matrix (V = 0.61, *p* = 0.002) (Figure 6). Substantial to almost perfect inter-rater agreement was observed (κ = 0.79–0.91 for binary variables; weighted κ = 0.85 for ordinal marginal seal). Surface roughness (Ra), defined as the primary outcome, was significantly lower in the MMT group compared to R2T (*p* < 0.001, 95% CI) (Figure 7). The effect size was large (δ = −0.88), indicating a substantial (3-fold) reduction in surface roughness when using the matrix-based technique.

## 4. Discussion

In order to restore deep subgingival defects predictably, the practitioner has a number of proven options at their disposal. Surgical crown lengthening and intentional replantation are associated with increased morbidity due to surgical intervention, but can yield reproducible results. Surgical crown lengthening involves sacrificing attachment in order to restore an adequately dimensioned, supracrestal connective tissue attachment. However, this may result in an unfavorable crown-to-root ratio. In addition, the risk of tissue relapse may be increased depending on the gingival phenotype [33]. Intentional replantation carries the risk of external resorption [34]. Although this risk may be mitigated by rapid orthodontic extrusion using rubber bands, magnets, or bracket systems, this approach requires weekly fibrotomy and is associated with increased treatment costs [35].

Another treatment option is the deep margin elevation described above, in which the proximal box is raised in a controlled manner using composite, thereby shifting the previously subgingival working area to a supragingival position. This approach significantly reduces challenges such as difficult contamination control, preparation, impression taking, and restoration delivery, as the working area can be kept absolutely dry using a rubber dam and becomes much easier to see and control. To bridge the time window in which absolute isolation is not possible, meticulous control using optical magnification, gingivectomy if necessary, hemostasis and an adapted filling technique are mandatory, as previous studies have shown that contamination by saliva and/or blood can significantly reduce adhesion values and lead to restoration failure [19,36]. The adhesive interface between the cavity floor and the raised increment (dentin–composite) is therefore crucial for treatment success. However, the technical sensitivity of this procedure also means that this form of treatment should be carried out by trained personnel or specialized practitioners. The interface between the raised box and the indirect restoration (composite-composite) can be bonded in a highly predictable manner and does not represent a weak spot [37,38]. This explains why DME techniques are highly compatible with etchable all-ceramic restorations [39]. In an era in which adhesive and CAD/CAM techniques are well advanced and well established, these treatment steps can also be performed in a single treatment session. Teeth that require a DME are usually severely damaged and/or have undergone endodontic treatment and are therefore more susceptible to fracture from a biomechanical point of view than vital teeth with small cavity classes [40]. Partial or full cusp coverage using all-ceramic onlays or overlays in combination with DME is a minimally invasive approach that aims to re-establish physiological force transmission and can reduce the risk of tooth fracture [41,42].

Since Magne and Spreafico described the modified matrix technique in 2007, many in vitro studies and case reports, several narrative and systematic reviews, and a few clinical studies have been conducted [25,28,43,44,45,46,47,48,49,50,51,52,53,54,55,56]. The key findings of these studies are that DME resulted in significantly less microleakage than deep indirect restorations without DME and that DME did not negatively affect surface integrity or fracture behavior. In addition, the elevation material and the adhesive system used for fixation appear to influence the marginal fit of the restoration. DME had no influence on fatigue behavior, fracture strength, failure pattern, or reparability. Since the DME procedures essentially involve the technique of “immediate dentin sealing” (IDS), DME can strengthen the adhesive bond between the indirect restoration and the tooth substrate [30]. This is because the freshly cut dentin is immediately sealed with dentin adhesive during the same session. Unlike a conventional protocol, this eliminates a temporary phase in which open dentin tubules are exposed to the oral environment or temporary material. By avoiding this contamination, the adhesive bond is improved and thus also the prognosis for the tooth. In addition, DME and subgingival restorations are compatible with periodontal health, provided they are well polished. However, the available literature is mainly limited to in vitro studies [51,54]. It is noteworthy that in the total of 133 studies included in the reviews [50,52,53,54], the comparison between MMT and matrix-free technique is not addressed. To substantiate this statement, a structured literature search was conducted in PubMed, Scopus, and Google Scholar up to February 2026. The following Boolean search strategy was applied: (“deep margin elevation” OR “margin relocation” OR “cervical margin relocation” OR “proximal box elevation” OR “modified matrix technique”) AND (“matrix-free technique” OR “R2-Technique” OR “freehand technique”). The aim of our experiments was therefore to investigate whether there was a difference in terms of roughness, surface integrity, marginal fit, and excess material depending on the technique chosen. Our experiments revealed that the primary null hypothesis, “DME technique does not influence surface roughness of the elevated composite increment”, could be rejected.

Since the modified matrix technique involves layering against a prefabricated smooth metal band, the average surface roughness of the composite increments in this group was significantly smoother than with the matrix-free technique. It is known from the literature that more pronounced surface roughness correlates with increased plaque accumulation and maturation [57,58]. It was shown that bacterial colonization increased significantly at a roughness depth of >2 µm. In our experiments, this threshold was exceeded with the matrix-free technique. However, whether this difference translates into clinically relevant plaque accumulation under intraoral conditions remains uncertain. The difference in surface roughness between the two techniques was also confirmed by SEM images (Figure 4B,C). It has been described that aluminum oxide-coated polishing discs leave the smoothest surfaces [59]. However, we deliberately avoided using polishing discs in our experiments, as polishing with these tools seems clinically unrealistic in a deep approximal box. These data do not permit conclusions regarding the clinical relevance of a 2 µm surface roughness difference. Older studies have shown, for example, that composite applied subgingivally showed no difference in plaque accumulation compared to untreated enamel [60]. It can be assumed that today’s composite materials are at least equivalent, but probably of enhanced quality. Furthermore, it has been proven that plastic matrices generate smoother composite surfaces than metal matrices [61]. However, since DME requires considerable traction and pressure, plastic matrices appear unsuitable, which is why we did not use them in our experiments.

Despite the smoother surfaces of the MMT, it is noticeable that overhangs and excess bonding occurred significantly more frequently with this technique. In terms of marginal fit, overhangs, and excess bonding material, the R2T showed advantageous results. Overhangs of proximal restorations correlate positively with periodontal tissue loss and should therefore be avoided at all costs in the dentogingival complex [62,63,64]. The favorable results are likely attributable to surface polishing beyond the defect margins. When looking at the scores for air inclusions and bubbles, it is noticeable that the MMT performs objectively worse. These inclusions probably occur when the flowable is applied against the matrix. In this case, it may be helpful to use a probe to spread the composite or to work with different viscosities using the snowplow technique [29]. We used flowable composite because this material performed better in individual studies and reviews with regard to marginal seal and adaptation in DME [53]. A self-etching single-bottle adhesive was selected because, in a clinical setting, it is essential to keep this delicate treatment step as short as possible in order to reduce the risk of contamination. A time-consuming multi-bottle adhesive can be disadvantageous in this context. The etch-and-rinse mode of a self-etching single-bottle adhesive was selected even though phosphoric acid etching on clean dentin is not strictly necessary [65]. In everyday clinical practice, however, hemostatic agents (e.g., iron III sulfate or aluminum chloride) are often used to stop gingival bleeding by coagulation and clogging capillary openings. Since these materials are quite fluid and difficult to control, they often coat the adhesive surface and must be considered as contamination. It has been shown that contamination with hemostatic agents dramatically reduced adhesive bond strength, as self-etching single-bottle adhesives alone were not able to counteract contamination [66,67,68]. However, the initial bond values could be re-established by briefly etching the dentin and adequately decontaminating it with water spray [69,70]. For this reason, we also used a 10-s phosphoric acid etch followed by a 15-s water spray in our experiments. The short etching time was chosen to avoid “over-etching” and thus the collapse of the collagen network [71].

The requirements for the optimal subgingival restoration material are high: biocompatibility, no solubility, radioopacity, compactness, hardness, smooth surfaces, polishability, easy handling, no shrinkage or expansion, predictable, easy polymerization, perfect marginal fit, no microleakage, etc. [72,73]. Unfortunately, none of the materials available today meets these requirements; however, highly polishable bulk-fill composites come quite close. Dental ceramics (e.g., lithium disilicate) fulfill most of the requirements, but require a meticulously controlled working environment due to their technique-sensitive bonding. In order to serve as a foundation for indirect restorations, a deeper-lying composite, which is used for DME, must also meet these requirements in particular. Various studies have shown that composites with smooth, well-adapted surfaces can be tolerated by the organism subgingivally and even close to the bone crest [74,75]. In a randomized, controlled clinical study conducted in 2016, severely decayed teeth were treated using DME or surgical crown lengthening and compared in terms of probing depth, bleeding on probing, and plaque index [76]. It was found that the two groups did not differ significantly from each other, which suggests that DME was well tolerated. In another clinical/histological study, patients who underwent DME were followed up and periodontal parameters such as probing depth and BOP were analyzed [77]. The study revealed that subgingival restorations were compatible with periodontal health and showed similar values to untreated root surfaces, even though the follow-up period was only 3 months. These studies provide preliminary evidence that DME should be considered a useful alternative or supplement to conventional resective therapy. However, there is also scientific evidence that damage to the supracrestal connective tissue attachment can lead to attachment loss, inflammation, suppuration, or recession, depending on the gingival phenotype [24,26,78]. If such damage is limited to a very small area or only to the epithelial attachment, and if well-adapted and smooth surfaces are created, these often remain inconspicuous in everyday clinical practice and are compatible with gingival and/or periodontal health [29,53]. Sahrmann et al. were able to show in a meta-analysis that the extent of epithelial attachment is subject to individual and tooth-related variations [79]. To maintain healthy conditions, both adequate oral hygiene on the part of the patient and regular follow-up appointments are necessary [51]. Smooth, well-adapted surfaces as well as anatomically shaped proximal contacts and emergence profiles are extremely important for hygiene and accessibility [63] and can be established with the help of DME techniques. This is particularly significant given that genuine periodontal attachment cannot form in the area of the DME, as Sharpey’s fibers naturally require root cement into which they can insert. However, epithelial attachment can occur on smooth surfaces but is characterized by a narrower area of supracrestal connective tissue attachment and longer junctional epithelium.

Referring to methodological considerations and limitations of this study, it should be noted that the study was conducted under strictly standardized in vitro conditions, allowing for exact control over variables such as cavity size, adhesive protocol, and composite placement to ensure reproducibility and comparability of the two techniques. In vivo conditions, such as moisture contamination, patient-related anatomical variability, limited access, and operator fatigue, may influence the performance and outcomes of both techniques. Furthermore, the teeth used were extracted human molars with idealized, standardized defects. This does not reflect the full clinical spectrum of deep proximal lesions, which often present irregular shapes and patient-specific challenges. Furthermore, the score system was developed to allow structured assessment of marginal and structural integrity based on the number of transition surfaces exhibiting gap-free adaptation. This approach is conceptually consistent with established SEM-based evaluations of marginal continuity, which assess the presence or absence of gap-free margins [80]. All score levels were defined a priori using operational criteria and evaluation was carried out by two blinded and independent examiners to ensure reproducibility. Reproducibility was confirmed by substantial to almost perfect inter-rater agreement. Future studies may further validate this structured classification approach. The polishing protocols used for the matrix-free technique may also not always be feasible clinically, especially in posterior regions with limited access. Another limitation is the evaluation of outcomes predominantly by SEM and optical profilometry, which—despite their precision—cannot represent the biological response of the surrounding periodontal tissues. While surface smoothness, marginal adaptation, and absence of voids are important parameters, clinical endpoints such as inflammation, bleeding on probing, and patient satisfaction could not be assessed in this setting. Therefore, while this study provides important insights into the morphological and technical outcomes of two DME techniques, further randomized, controlled clinical trials are necessary to validate these findings under real conditions and to assess their long-term biological compatibility.

MMT enables controlled composite layering against a rigid matrix and may be advantageous in anatomically favorable situations where stable matrix adaptation can be achieved, especially along straight proximal defects. An initial attempt with MMT may therefore be useful if predictable matrix sealing appears possible. In complex anatomy, such as pronounced proximal concavities or the furcation roof, rigid matrix adaptation can lead to incomplete sealing of the box floor. If a visible gap remains despite the use of wedges or PTFE tape, switching to the R2T technique may be advisable to avoid uncontrolled excess bonding and/or composite material, which, according to our in vitro experiments, occurred more frequently in association with MMT. Overall, both techniques may be clinically applicable when performed carefully. The choice of technique should be made individually based on anatomical conditions (straight vs. irregular shape), accessibility, and user preference. These considerations are pragmatic recommendations derived from laboratory findings and do not constitute definitive treatment guidelines.

## 5. Conclusions

In summary, DME can be a valuable tool for predictably preserving even deeply subgingivally destroyed teeth. Within the limitations of this in vitro study, both techniques demonstrated distinct and technique-related characteristics. Our experiments showed that the MMT resulted in significantly smoother surfaces, which may influence plaque retention, although clinical relevance cannot be inferred from the present in vitro data. However, our SEM images also showed that this technique resulted in significantly more bonding excess and increased void formation. Although the R2T resulted in rougher surfaces, it led to significantly more homogeneous marginal adaptation and fewer overhangs. The primary null hypothesis could be rejected. A definitive judgment regarding the overall superiority of either technique cannot be made. Both methods may be clinically applicable when carefully performed, and their selection should be individualized based on anatomical configuration, accessibility, and operator preference. It should be noted that our experiments were in vitro experiments conducted under optimal dry-lab conditions. Further randomized controlled clinical trials are warranted to compare the techniques under real-world clinical conditions and to evaluate their long-term biological compatibility.

## Figures and Tables

**Table 1:** Scoring System.

Outcome	Description	Score
Roughness	Visually smooth surface	1
	Visually rough surface, grooves	2
Marginal seal	3 transition surfaces without gap	1
	2 transition surfaces without gap	2
	1 transition surfaces without gap	3
	0 transition surfaces without gap	4
Bubbles/air inclusions	No bubbles/air inclusions	1
	Bubbles/air inclusions	2
Overhangs	No overhangs	1
	Overhangs	2
Bonding-surplus	No excess bonding	1
	Excess bonding	2

## Data Availability

The datasets presented in this article are not readily available because of ongoing studies but are available from the corresponding author on reasonable request.

## Abbreviations

The following abbreviations are used in this manuscript:

CEJ	cementoenamel junction
CI	confidence interval
DME	deep margin elevation
IDS	immediate dentin sealing
MMT	modified matrix technique
OPMI	operating microscope
OR	Odds ratio
R2T	R2 technique
SA	supracrestal attachment
SEM	scanning electron microscopy
V	Cramér’s V
